# The Role of Fibrinogen, Homocysteine and Metabolic Syndrome’s Alterations in Sudden Sensorineural Hearing Loss (SSHL): A Narrative Review

**DOI:** 10.3390/medicina59111977

**Published:** 2023-11-09

**Authors:** Daniele Monzani, Carlotta Liberale, Erika Segato, Francesca De Cecco, Valerio Arietti, Silvia Palma, Luca Sacchetto, Riccardo Nocini

**Affiliations:** 1 Unit of Otorhinolaryngology, Head & Neck Department, University of Verona, Piazzale L.A. Scuro 10, 37134 Verona, Italy; daniele.monzani@gmail.com (D.M.); carlotta.liberale@gmail.com (C.L.); francesca16592@gmail.com (F.D.C.); valerio.arietti@gmail.com (V.A.);; 2 Otolaryngology and Audiology Unit, University of Modena and Reggio Emilia, 41100 Modena, Italy; silviapalma@inwind.it

**Keywords:** sudden sensorineural hearing loss, fibrinogen, homocysteine, metabolic syndrome, therapies

## Abstract

Fibrinogen and homocysteine (HCY) are molecules known to play a role in vascular homeostasis, and their blood levels are often elevated in patients with metabolic syndrome. Recent evidence suggests that sudden sensorineural hearing loss (SSHL) may have a vascular origin. This has led many authors to advocate that fibrinogen, homocysteine, and metabolic syndrome (MetS) may play a direct role in SSHL. The aim of this brief review is to examine the role and influence of these molecules and MetS on the mechanisms of SSHL. Elevated fibrinogen levels have been associated with a worse prognosis in SSHL, possibly due to increased blood viscosity and decreased blood flow. Similarly, HCY has been associated with vascular damage, particularly in hyperhomocysteinemia, although the exact association with SSHL remains controversial. MetS has been demonstrated to function both as a causative factor and as a contributor to poorer recovery in cases of SSHL. However, although some studies suggest a possible role for these biomarkers and MetS in the prognosis and treatment of SSHL, specific therapeutic and preventive strategies based solely on these factors have yet to be developed. Given their potential role in prognosis and treatment and the global epidemic of metabolic syndrome, this issue needs to be analyzed comprehensively. Thus, further quality studies need to be conducted, even though it is difficult to determine the actual impact of MetS on the development of SSHL, as it is a multifactorial disease affecting multiple organs.

## 1. Introduction

Sudden sensorineural hearing loss (SSHL) is defined as hearing loss occurring within 72 h with a loss of at least 30 decibels in three adjacent frequencies (The American Academy of Otolaryngology—Head and Neck Surgery) [1]. The incidence of SSHL is estimated to be 5–20 cases per 100,000 [2], but the actual incidence may exceed these estimates because individuals who experience rapid recovery from the disease often do not seek medical attention [3]. The exact cause of SSHL is unclear in many cases [4], but several potential factors and conditions have been associated with its development. The cause of SSHL can be determined in only 10 to 15% of patients [5]. Possible causes and contributing factors for sudden hearing loss include viral infections, vascular problems, autoimmune diseases, medications, trauma, tumors, neurological diseases, and genetic predisposition [6]. Some authors have suggested that idiopathic SSHL may have a vascular origin, indicating that a local ischemic event leading to hypoperfusion of the cochlea may play a pathogenetically relevant role. The reason why the cochlea appears so vulnerable to vascular insults is to be found in its vascularization: the cochlea is supplied with blood by two small terminal arteries that are small in diameter and lack a collateral blood supply [7]. It is interesting to notice how unilateral SSHL has a clinical presentation comparable to ischemic vascular diseases such as amaurosis fugax and transient ischemic episodes [8].

Numerous treatment options have been explored for idiopathic SSHL, including anti-inflammatory agents, antimicrobial drugs, calcium antagonists, vitamins, essential minerals, vasodilators, volume expanders, defibrinogenating agents, diuretics, hyperbaric oxygen therapy, and bed rest [5], but the chances of recovery from idiopathic hearing loss depend on the severity of hearing loss, duration, and age at presentation [6].

The current Clinical Practice Guideline for Sudden Hearing Loss recommends that systemic steroids and/or hyperbaric oxygen therapy (HBOT) may be considered as treatment options if administered within two weeks of symptom onset. However, it is important to note that the efficacy of these treatments may vary greatly from person to person, possibly due to the different causes of SSHL. Therefore, the routine use of antiviral drugs, antithrombotics, vasodilators, vasoactive agents, and similar therapies is not recommended for the standard treatment of SSHL [9]. It is important to emphasize that clinical trials of drugs for the treatment of SSHL are difficult to conduct because of the difficulty in identifying control groups and because many factors may affect the prognosis of patients [10]. Therefore, there remains limited consensus and no clear direction based on the currently available evidence on the molecules or biomarkers involved in SSHL.

According to recent evidence, fibrinogen and homocysteine play a role in sudden sensorineural hearing loss (SSHL). Also, MetS, with its intrinsic biochemical alterations, has been shown to have the potential to cause vascular damage, even within the cochlea. Consequently, the inflammatory mechanisms within the vascular network may lead to cochlear cell damage, contributing to the development of SSHL. Understanding these factors may improve our understanding of the vascular and circulatory aspects of SSHL, which may allow the development of more targeted diagnostic tools and therapeutic interventions. Such insights may ultimately lead to better outcomes for patients with SSHL, giving them a better chance of preserving their hearing function and overall quality of life.

In this brief narrative review, we aim to examine the role and influence of these factors on the mechanisms of SSHL. Investigating the role of fibrinogen, homocysteine, and MetS in sudden sensorineural hearing loss (SSHL) is of paramount importance because of the potential impact on both the diagnosis and treatment of this debilitating condition.

## 2. Materials and Methods

To collect relevant scientific literature on our research topic, we conducted a comprehensive search through the PubMed database. The search included articles published within the last 25 years. The following strings and keywords were used in our search strategy:

1. “fibrinogen” AND “Sudden sensorineural hearing loss (SSHL)”;
2. “homocysteine” AND “Sudden sensorineural hearing loss (SSHL)”;
3. “Metabolic Syndrome” AND “Sudden sensorineural hearing loss (SSHL)”.

The search was carried out to identify studies and articles that explored the relationships and interactions between these keywords, as they pertain to our research objectives.

The included articles had to meet the following criteria:

- Published within the last 25 years (from 1998 to 2023);
- Written in English;
- Focused on the association between fibrinogen, homocysteine, metabolic syndrome, and sudden sensorineural hearing loss;
- Available as full-text articles.

Excluded articles were those that did not meet these criteria or were duplicates of other retrieved publications.

## 3. Discussion

Sudden sensorineural hearing loss (SSHL) is a medical emergency that requires immediate ENT medical attention. Much of the literature indicates that 32% to 65% of cases of SSHL can recover spontaneously. However, clinical experience indicates that these numbers may be overestimated [11]. Various hypotheses have been proposed in the scientific literature to explain sudden sensorineural hearing loss (SSHL), although specific triggers can only be described for a relatively small proportion of SSHL cases, ranging from 10% to 30% [12]. In fact, it is estimated that up to 90 percent of cases of SSHL are idiopathic at presentation, and approximately 1% to 3% of SSHL cases can be attributed to retrocochlear disorders, which may include conditions such as acoustic neuromas, meningiomas, demyelinating diseases, or strokes [13].

According to a review of the evidence regarding the etiology of sudden sensorineural hearing loss (SSHL) in adults conducted by Chau et al. [14], they discovered that the suspected etiologies included idiopathic (71.0%), infectious diseases (12.8%), otologic diseases (4.7%), trauma (4.2%), vascular or hematologic issues (2.8%), neoplastic causes (2.3%), and other factors (2.2%). The establishment of a direct causal link between SSHL and these etiologies remains elusive. The low incidence of vascular or hematologic issues could be related to the challenges in demonstrating a causal-effect link. In fact, there is still no established method for detecting vascular damage in the cochlea, and it’s possible that several idiopathic cases could be related to vascular issues.

Several lines of research have focused on the concept that altered blood flow and/or oxygenation lead to cochlear hair cell damage and hearing loss [15]. According to the vascular theory, hearing loss may be caused by various factors such as sudden vascular hemorrhage, blockage due to emboli, vasospasm, changes in blood viscosity, and other risk factors including hypercholesterolemia, hyperfibrinogenemia, and microembolism [9]. This suggests that a localized ischemic event resulting in reduced blood flow to the cochlea could be a significant factor in its pathogenesis. In a review by Doo et al., the authors examined various hemostatic parameters and tested the hypothesis that certain blood molecules may play a role in altering blood flow, particularly in the labyrinthine artery [10].

**Figure 1:**
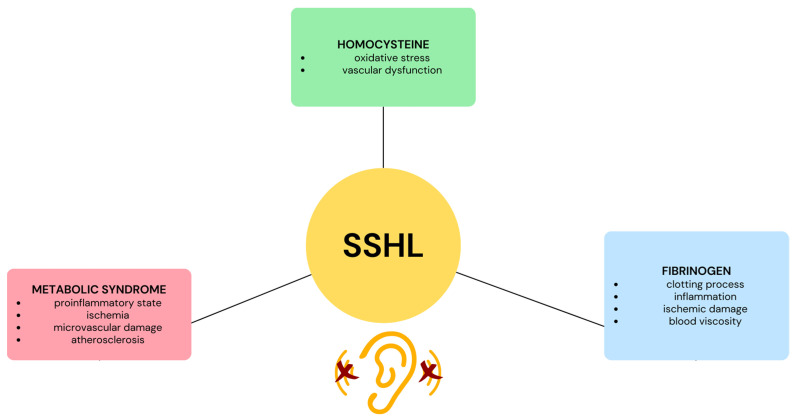
Illustration depicting the influence of fibrinogen, homocysteine, and metabolic syndrome on sudden sensorineural hearing loss (SSHL).

Given the theory of microvascular occlusion, it is worthwhile to investigate hypercoagulability in patients with idiopathic SSHL, especially when thrombophilia-related abnormalities or predominant venous factors such as oral contraceptive use, pregnancy or puerperium, and cardiovascular factors such as arterial hypertension, hyperlipidemia, diabetes, and cigarette smoking are present. Thrombophilia abnormalities can be divided into two categories: inherited and acquired. Inherited thrombophilia includes conditions such as deficiency of natural anticoagulant proteins such as antithrombin, protein C, and protein S, and gain-of-function mutations in the genes of factor V and prothrombin. On the other hand, acquired thrombophilia is due to factors such as the presence of antiphospholipid antibodies. In addition, there are two other thrombophilia-related disorders for which there is no clear evidence of inheritance. These are elevated plasma levels of coagulation factor VIII and mild to moderate hyperhomocysteinemia (HHcy). Finally, circulating microparticles, tiny phospholipid-rich particles released by various blood cells such as platelets, endothelial cells, leukocytes, and erythrocytes, are also considered potential risk factors for sudden sensorineural hearing loss (SSHL) because they are associated with an increased risk of thrombosis [15] (Figure 1).

### 3.1. Fibrinogen

Fibrinogen is a glycoprotein normally present in human blood plasma. It is mainly produced by the liver and released into the bloodstream. It plays a central role in several biological functions, including hemostasis and inflammation [16]. Although fibrinogen exists as a soluble macromolecule, it undergoes a crucial transformation in response to vascular injury. Through a series of enzymatic reactions, it is converted to fibrin, forming a mesh-like structure that effectively traps blood cells and facilitates the formation of a stable clot at the site of injury. This clotting process is important for preventing excessive bleeding and maintaining the integrity of the circulatory system. In addition, the involvement of fibrinogen in inflammation is noteworthy, as it interacts with various molecules and cells, contributing to the body’s response to injury and infection [17]. Fibrinogen may suggest a link between vascular factors and SSHL and shed light on underlying mechanisms.

In a study in guinea pigs, an association was found between elevated serum fibrinogen levels and a decrease in blood flow in the cochlea. It was also observed that administration of a defibrinogenating drug improved blood flow in the cochlea [18]. In a prospective cohort study by Okada [19] et al., the authors investigated the prognostic significance of fibrinogen and inflammatory cytokines in sudden sensorineural hearing loss (SSHL). The study revealed a remarkable inverse relationship between SSHL recovery rate and serum fibrinogen level. This decrease in blood flow, due to thrombosis in the inner ear, leads to oxygen starvation and apoptosis of the hair cells of the cochlea. Consequently, individuals with elevated fibrinogen levels, potentially indicative of ischemic damage to the cochlea, have a limited response to treatment, independent of concurrent inflammatory conditions. These findings underscore the role of ischemic changes in the inner ear as a possible cause of SSHL, ultimately leading to a suboptimal response to steroid therapy.

Indeed, fibrinogen levels may indicate increased blood viscosity, which in turn could lead to decreased blood flow in the affected regions. In 2018, Oya et al. performed a literature review on the relationship between SSHL and fibrinogen and analyzed 1577 patients. They found that a high serum fibrinogen level correlated with a poor prognosis [4]. These results were also confirmed by Kanzaki et al. [20]. Moreover, they suggested that hyperfibrinogenemia may not be directly related to the cause of SSHL, but rather to the severity of the disease. Also, Sun et al. [21] in 2023 found that blood fibrinogen level is a reliable indicator of the prognosis of SSHL when all frequencies are affected. In addition, recent studies have confirmed that thrombosis occurring in the inner ear can lead to a reduction in blood flow. This reduction may ultimately lead to inadequate oxygenation and apoptosis of the hair cells of the cochlea. Therefore, thrombosis appears to be the most likely cause of SSHL at all frequencies.

As noted by Hira et al., plasma fibrinogen levels increase susceptibility to atherosclerosis due to two main factors: increased plasma viscosity and thrombotic activation triggered by fibrin during its degradation [22]. Numerous studies have indicated that this mechanism may lead to idiopathic sudden hearing loss (SSHL) when it occurs in the inner ear. Oreskovic et al. investigated the prevalence of abnormal lipoprotein and fibrinogen levels in patients with SSHL [23]. They suggested that these findings could serve as a basis for developing new treatments or prevention strategies in the future [23]. In a recent literature review by Linthicum [13], the authors recommend a complete blood count and coagulation test (fibrinogen) for all cases of sudden SSHL. Thus, these tests are inexpensive, readily available, and provide as much information about the diagnosis and prognosis of sudden SSHL as any other biomarker.

However, it is important to note that the pathophysiology of SSHL at both low and high frequencies is not yet confirmed to be due to only thrombosis. Consequently, the role of fibrinogen remains unclear, and its impact on prognosis is currently not fully understood. To date, it is not possible to definitively determine whether hyperfibrinogenemia directly causes SSHL. In fact, it remains unclear whether hyperfibrinogenemia is the cause or the consequence of SSHL [4].

### 3.2. Homocysteine

Homocysteine (HCY) is an amino acid produced in the body as a byproduct of the metabolism of another amino acid called methionine. It is also a homolog of cysteine [24]. Several factors can lead to an increase in HCY levels, including genetic influences, dietary habits, lifestyle factors, certain medications, and more.

A laboratory study showed that when homocysteine levels are elevated, a condition known as hyperhomocysteinemia (hHCY) can be associated with an increased risk of neurovascular disease, dementia, migraine, developmental disabilities, and epilepsy [25]. Mechanisms underlying the neurotoxic effects of HCY include oxidative stress, DNA damage, protein thiolation, and protein homocysteinylation, which may ultimately trigger processes such as apoptosis and excitotoxicity [24]. Elevated homocysteine levels, in turn, have been associated with impaired blood flow and vascular dysfunction, possibly contributing to SSHL.

As mentioned above, HCY is a risk factor for increased vascular injury, and its abnormal increase impairs endothelial function, reduces vascular flexibility, and leads to microvascular dysfunction, resulting in ischemia and hypoxia-related changes [26]. Currently, two main factors are widely recognized for the development of hyperhomocysteinemia: (I) nutritional elements characterized by a deficiency of metabolic cofactors such as vitamin B6, vitamin B12, and/or folic acid, and (II) genetic factors, including mutations in genes such as MTHFR and CBS, which may reduce enzyme activity. These factors may contribute to the accumulation of HCY in the body [27].

A retrospective case–control study by Passamonti et al. showed that HCY was associated with an increased risk of idiopathic SSHL, and hyperhomocysteinemia was reported as a negative prognostic factor for hearing recovery [12]. In addition, Fasano et al. found that HCY levels were higher in severe SSHL than in mild SSHL, demonstrating that HCY is associated with the degree of deafness in SSHL, as has been found for fibrinogen [4,28]. In a recent study, Wang et al. investigated the factors involved in the development of bilateral sudden sensorineural hearing loss. They focused specifically on HCY and confirmed the evidence found by Passamonti et al. and Fasano et al. [29].

Niu et al. conducted a literature review on the association between SSHL and HCY in 2023 [30]. The study included nine articles and found that serum/plasma concentrations of HCY were higher in SSHL patients than in the control group. According to the results, elevated serum or plasma levels of HCY could be a risk factor for SSHL. They also suggest that vascular factors could be one of the causes of SSHL. To confirm the theory that hyperhomocysteinemia is a risk factor for SSHL due to vascular damage in the cochlea, further studies on pathophysiology are needed [30].

There are also some authors who do not believe in the role of HCY in SSHL. Cadoni et al. found no correlation between these two factors [31]. Also, Berner et al. conducted a study with 91 Danish adults and found no correlation between serum levels of folic acid, vitamin B12, and HCY and the occurrence of hearing loss [31]. Furthermore, in a cross-sectional study, Gopinath et al. reported that serum levels of folic acid, vitamin B12, and HCY were not significantly related to sudden hearing loss. However, they found that serum levels of folic acid and HCY were associated with age-related hearing loss [32]. Regarding the clinical effects of HCY on SSHL patients, Huang et al. conducted a study to investigate the association between plasma HCY concentration, serum folic acid concentration, and treatment occurrence and response in adult patients with total frequency deafness. The aim of this study was to test the hypothesis that higher plasma HCY levels, in association with lower serum folic acid levels, may be associated with a higher risk of sudden total frequency deafness [27]. Indeed, the researchers found that plasma HCY levels were elevated, and serum folic acid levels were decreased in patients with sudden hearing loss compared with the normal control group, with statistically significant differences (*p* < 0.05). Huang et al. attempted to administer folic acid, vitamin B6, and B12 to patients with SSHL, but their results showed no improvement in clinical outcomes. Nevertheless, they suggest that maintaining low levels of HCY in daily life may prove beneficial for the clinical prevention and treatment of hearing loss [27].

To date, there is insufficient evidence to definitively clarify the exact relationship between fibrinogen and HCY levels and their effects on hearing loss. Although many studies postulate this hypothesized relationship, this is not sufficient to develop therapeutic and preventive strategies based solely on these assessments.

### 3.3. Metabolic Syndrome (MetS)

While metabolic syndrome (MetS) itself does not involve single molecules like fibrinogen and homocysteine (HCY), it plays a significant role in causing various molecular dysfunctions that can potentially lead to vascular damage, which, in turn, affects the cochlea. As a condition with a high global prevalence, it is valuable to comprehend its implications, including a comprehensive analysis of its individual components. In this study, our objective is to assess the influence of metabolic syndrome (MetS) and the accompanying molecular alterations on the pathophysiology of sudden sensorineural hearing loss (SSHL).

Metabolic syndrome (MetS) is a set of metabolic disorders that include central obesity, insulin resistance or impaired glucose uptake and utilization, dyslipidemia, and hypertension [33]. Metabolic syndrome (MetS) has been defined as when a person has three or more of the following criteria. (1) Increased waist circumference (EWC): waist circumference ≥ 102 cm in men and ≥88 cm in women; (2) increased blood pressure: blood pressure ≥ 130/85 mmHg or drug treatment for previously diagnosed hypertension; (3) decreased HDL-C: <40 mg/dL in men and <50 mg/dL in women or specific treatment for decreased HDL-C; (4) increased TGs: TG level ≥ 150 mg/dL or drug treatment for elevated TG; and (5) elevated fasting glucose: fasting glucose level of ≥100 mg/L or drug treatment for elevated glucose and previously diagnosed type 2 diabetes [34].

Its multisystemic pattern arises from a confluence of factors including pro-inflammatory states, oxidative stress, hemodynamic dysfunction, and ischemia in ‘dysmetabolic’ patients. This convergence leads to the emergence of various clinical conditions, encompassing cardiovascular diseases (CVD), non-alcoholic steatohepatitis, liver dysfunctions, chronic kidney disease, numerous types of cancer, and neurodegenerative disorders [35].

Metabolic syndrome and its associated components have the potential to lead to several comorbidities, one of which is inner ear dysfunction. The exact mechanism linking metabolic syndrome and hearing loss is not yet clear, but there is evidence that it may be related to peripheral blood vessel dysfunction [36]. It is known that subjects with metabolic syndrome had significantly higher plasma fibrinogen and homocysteine levels than subjects without metabolic syndrome. This could contribute to damage to the vascularization of the cochlea, as the primary mechanism responsible for hearing loss appears to be vascular damage within the cochlea [37]. Chien et al. reported that metabolic syndrome increases the risk of SSNHL by 3.5-fold [38]. These findings were confirmed by a study by Park et al. [39], which found that metabolic syndrome was more prevalent in SSNHL patients and that patients with metabolic syndrome responded worse to treatment than patients without this syndrome.

Metabolic syndrome encompasses a number of disorders that include insulin resistance and abnormal fat deposition. The combination of these factors can lead to microvascular damage and endothelial dysfunction [40]. Numerous studies in the existing literature have investigated the relationship between metabolic syndrome and recovery from sudden sensorineural hearing loss. However, there is a notable lack of studies directly investigating metabolic syndrome as a primary cause of SSHL. Zand et al. conducted a study that specifically looked at the causal relationship between MetS and SSHL [40]. A study by Chien et al. found that metabolic syndrome plays a role in the development of idiopathic SSNHL but has no impact on the prognosis of idiopathic SSNHL [38]. The study by Zand et al. highlighted that both insulin resistance, which seems to be the main cause of metabolic syndrome, and hyperlipidemia cause atherosclerotic changes and endothelial dysfunction, leading to cochlear microangiopathy [40]. Hence, SSHL in patients with metabolic syndrome (MetS) appears to be associated with a range of molecular alterations. These alterations involve factors such as fibrinogen, HCY, hyperlipidemia, and elevated blood glucose levels. Currently, it is still not possible to definitively determine which molecule plays a decisive role in the pathophysiology of SSHL. Therefore, metabolic syndrome (MetS) should be regarded as a complex entity.

MetS has also been shown to be associated with poorer recovery in SSHL. Indeed, metabolic syndrome had a negative impact on hearing improvement in patients with idiopathic SSNHL. Lower initial hearing threshold, absence of diabetes mellitus and hypertension, and BMI < 25 were associated with improvement in hearing after treatment, according to the results of Zand et al. [40]. These findings were confirmed by Jung et al., who conducted a study showing that individuals with metabolic syndrome had lower rates of complete and partial improvement, while the rate of no improvement was higher. This study provides further evidence of the negative impact of metabolic syndrome on the prognosis of patients with sudden sensorineural hearing loss [41]. The authors also discovered that correction of dyslipidemia in patients with SSHL in the chronic phase led to an improvement in hearing, providing further evidence of the association between dyslipidemia and the occurrence and prognosis of hearing impairment [41]. Jung et al. emphasized that hypertension is a risk factor for SSNHL. People with hypertension are more likely to develop SSNHL and have a lower cure rate than people without hypertension. Elevated blood pressure contributes to decreased elasticity of blood vessels in the inner ear, leading to atherosclerotic changes that cause narrowing of the vessels, decreased blood flow, and increased damage to the cochlea. It has been widely demonstrated in the literature that microangiopathy is a pathological mechanism associated with all five factors of metabolic syndrome, as well as vascular mechanisms that are one of the causes of SSHL. To date, the metabolic syndrome appears to play a dual role in the development of SSHL. On the one hand, it contributes to vascular damage that directly affects the cochlea; on the other hand, it leads to poorer recovery from SSHL, slows down the healing process, and impairs the improvement of hearing.

## 4. Conclusions

Numerous studies have addressed the involvement of blood parameters, particularly fibrinogen and homocysteine, in the context of SSHL. Elevated fibrinogen levels have been associated with a poorer prognosis, possibly due to increased blood viscosity and decreased blood flow. Similarly, HCY has been associated with vascular damage, particularly in cases of hyperhomocysteinemia, although the exact association with SSHL remains controversial.

While recent findings have shed light on the possible involvement of fibrinogen and HCY in the pathophysiology of SSHL, concrete data confirming their direct link to cell damage are still lacking. Moreover, their role in the prevention or treatment of SSHL remains unclear. To date, they remain hypothetical factors within a complex mechanism involving several elements. Several studies have investigated metabolic syndrome (MetS) and all its molecular alterations as a possible cause of sudden sensorineural hearing loss because of its impact on the vascular network and endothelial damage, particularly in the cochlear region. Indeed, MetS has been shown to affect the recovery process of SSHL, with patients with elevated levels of MetS parameters showing poorer recovery outcomes. Further research is needed to gain a comprehensive understanding of this complicated issue.

## 5. Future Directions

The clinical implications of HCY, fibrinogen, and molecular alterations of MetS in SSHL remain a focus of research. Given their potential role in prognosis and treatment and the global epidemic of metabolic syndrome, this issue needs to be analyzed comprehensively. The actual pathogenetic role in sudden sensorineural hearing loss is not yet fully understood, so specific therapeutic and preventive strategies based solely on these biomarkers have yet to be developed. In the future, comprehensive and multidimensional research approaches should be prioritized. First, researchers should consider conducting large-scale, longitudinal cohort studies to establish causal relationships and describe the precise mechanisms by which fibrinogen, homocysteine, and molecular alterations of metabolic syndrome affect auditory function. In addition, investigating potential synergistic effects between these factors and other risk factors, such as age, genetics, and lifestyle, would provide a more holistic understanding of their impact on hearing loss. In addition, the incorporation of advanced imaging techniques such as functional MRI or Doppler ultrasound could help visualize the vascular changes associated with fibrinogen and homocysteine, providing valuable insights. Finally, interventional studies examining the efficacy of therapies to prevent or treat hearing loss by affecting fibrinogen and homocysteine levels could provide valuable clinical recommendations for future treatment strategies. With these multifaceted approaches, future studies can contribute significantly to our understanding of the role of fibrinogen, homocysteine, and metabolic syndrome in hearing loss and potentially lead to more effective prevention and treatment strategies. In addition, as recent studies have shown, there is increasing evidence of biomarkers for the prognosis of SSHL. Heat shock protein-70, anti-endothelial cell antibodies, and prestin have shown potential in this regard. Therefore, further research on various blood molecules is needed to elucidate the specific mechanisms and prognosis of SSHL. This endeavor promises to pave the way for new targeted therapies and improve the efficacy of drugs against SSHL.

## Data Availability

The data supporting the findings of this paper are available and can be provided if requested.
